# The attitudes of nonpsychiatric nurses towards mental disorders in China

**DOI:** 10.3389/fpsyt.2024.1420632

**Published:** 2024-06-27

**Authors:** Qi-Kai Wang, Xu Wang, Ya-Jing Qiu, Wen-Xin Bao, Xia-Can Chen, Jia-Jun Xu

**Affiliations:** ^1^ Mental Health Center, West China Hospital of Sichuan University, Chengdu, China; ^2^ West China School of Basic Medical Sciences and Forensic Medicine, Sichuan University, Chengdu, China

**Keywords:** social stigma, mental health, nursing education research, nurses, China

## Abstract

**Background:**

Few studies have explored the associated factors of attitudes of nonpsychiatric nurses towards mental disorders. Therefore, this study is aimed to evaluate the attitudes of nonpsychiatric nurses towards mental disorders and especially explore the association between psychiatric clinical practice and these attitudes.

**Methods:**

A total of 1324 nonpsychiatric nurses and students majoring in nursing were recruited through an online questionnaire from December 2021 to March 2022 in Sichuan Province, China. Demographic information, personal care experience, psychiatric nursing education and the Community Attitudes towards the Mentally Ill (CAMI) were collected. A higher score indicates a stigmatizing attitude in the authoritarianism and social restrictiveness (SR) subscales and a positive attitude in the benevolence and community mental health ideology (CMHI) subscales. Multivariate linear regression was employed to analyze associated factors of attitudes towards mental disorders, and hierarchical linear regression was used to analyze the association between psychiatric clinical practice and the attitudes towards mental disorders.

**Results:**

Under the control of confounders, high education level, long residence in urban and personal care experience were positively correlated with score of authoritarianism and SR (*p* < 0.05), and negatively correlated with score of benevolence (*p* < 0.05). Long residence in urban and personal care experience were negatively correlated with score of CMHI (*p* < 0.05). Hierarchical linear regression analysis showed that after adjusting for demographic information, psychiatric clinical practice was associated with lower score of benevolence (B = -0.09, 95%CI = -0.17 ~ -0.003, *p* = 0.043) and CMHI (B = -0.09, 95%CI = -0.17 ~ -0.01, *p* = 0.027), but the initial associations between psychiatric clinical practice and authoritarianism, SR disappeared.

**Conclusions:**

High education level, long residence in urban, personal care experience and the psychiatric clinical practice were associated with the discrimination of nonpsychiatric nurses towards mental disorders. Further exploring practical strategies to optimize the psychiatric clinical practice experience of nonpsychiatric nurses could help improve their attitudes towards mental disorders.

## 1 Introduction

Mental disorders are common worldwide, and their incidence has been rising globally (1). According to a recent national survey (2), the estimated 12-month prevalence and lifetime prevalence of mental disorders in China were 9.3% and 16.6%, respectively. The long and recurrent course of mental disorders leads to the shortening of people’s lifespans and the decline of their quality of life (3). Early detection and timely intervention from mental health professionals are crucial to improve the prognosis of mental disorders. As the group with the most direct and longest contact with patients in mental health services, nurses play a key role in both parts. Therefore, as a part of the healthcare front role, whether nurses discriminate against mental disorders or not could affect the patients’ further medical behavior, which in turn affects the early diagnosis and treatment.

The stereotypes, prejudice and discrimination towards mental disorders were stigma of mental disorders, which were often reflected through behavior of avoidance and social distance (4, 5). Mental health stigma poses a significant challenge on early diagnosis, timely treatment, treatment compliance, recovery of mental disorders and so on (6, 7). Recent researches have revealed that nursing students may regard mental disorders as unpredictable, potentially dangerous, and untreatable (8, 9). Therefore, they may exhibit a preference for maintaining social distance from patients, which prevents patients from seeking mental health services (10, 11). The nurses’ attitudes towards mental disorders have a significant impact on their behavior of coping with mental disorders, which in turn affect help-seeking behavior of patients (12). However, most patients with psychiatric symptoms initially seek help from nonpsychiatric professionals in general hospitals rather than psychiatrists (13). Therefore, as the gatekeepers to the mental health service by influencing the help-seeking behavior of patients, whether the nonpsychiatric nurses receive standardized training is vital for how to guide psychiatric patients to seek for mental health services.

Previous studies explored the attitudes of psychiatric nurses towards mental disorders and found that the attitudes of psychiatric nurses to patients with mental disorders were basically as negative as those of the general population, but were significantly more negative and stereotypical than those of other mental health professionals, such as psychiatrists, psychologists and social workers (14–16). In addition, a cross-sectional study noted that psychiatric nurses’ attitudes towards mental disorders were significantly associated with their clinical experience in mental health services, i.e., the more clinical experience they had, the more positive their attitudes towards mental disorders were (17). The results of these studies suggested that attitudes towards mental disorders might be related to the contents and forms of psychiatric education. Despite extensive research on psychiatric nurses’ attitudes, only a few studies have explored the attitudes of nonpsychiatric nurses towards mental disorders.

A cross-sectional study in Qatar found that nurses had more negative attitudes towards patients with mental disorders than doctors, and nonpsychiatric nurses had an even higher level of sigma than psychiatric nurses (18). However, another study on the attitudes of Finnish nonpsychiatric nurses showed positive attitudes (19). This evidence indicated that culture might contribute significantly to stigmatizing patients with mental disorders (20). Compared with Western culture, Chinese people emphasize “face culture” and interpersonal relationships, so stigma against mental disorders may be more common in Chinese society (21, 22). A recent survey in China has found that nonpsychiatric nurses working in general hospitals adopted stigmatizing attitudes towards mental disorders (23). However, the association between psychiatric clinical practice and the attitudes of nonpsychiatric nurses towards mental disorders has not been reported, so there is an urgent need to explore the association between psychiatric clinical practice and the attitudes of nonpsychiatric nurses towards mental disorders.

The purpose of China’s undergraduate level nursing education is to cultivate general nurses. Typically, undergraduate nursing students learn about mental disorders through two courses. The first is psychiatric nursing course, which covers the basics of mental disorders. Secondly, nursing students must complete clinical practice for no less than 40 weeks in the final year before graduation. They will rotate to different departments, including psychiatry nursing, where they can contact and participate in the clinical care of patients with mental disorders under the supervision of psychiatric nurses (24). There is evidence that contact with mental disorder, including direct contact, indirect filmed contact or educational email, can change nurses’ attitudes towards mental disorders in a positive way (25, 26). A study conducted in China found that having clinical practice experiences in psychiatric clinics contributed to improving nursing students’ attitudes towards mental disorders (21). Although professional contact (under the guidance of mental health professionals) with mental disorders of nonpsychiatric nurse is almost exclusively in psychiatric nursing education, nonpsychiatric nurses may be involved in the care of mental disorders at a personal level (e.g. family members, friends, etc.), which may also have an impact on their attitudes towards mental disorders (27). However, few studies explore the association between these factors and the attitudes towards patients with mental disorders concerning Chinese nonpsychiatric nurses.

Given these backgrounds, in this study we aim to evaluate the attitudes of nonpsychiatric nurses towards mental disorders, identify associated factors of attitudes towards mental disorder and further explore the association between psychiatric clinical practice and the attitudes of nonpsychiatric nurses towards patients with mental disorders.

## 2 Methods

### 2.1 Participants

This was a multicenter cross-sectional study investigating attitudes towards mental disorders among nonpsychiatric nurses by using multistage sampling. To avoid bias in single point sampling, cluster random sampling was adopted to separately select 2 public nursing schools and 1 hospital from 25 public nursing schools and 13 hospitals by lottery in Chengdu and Mianyang, Sichuan Province, China. Participants were recruited by convenience sampling from these institutions. Eligible participants were (1) people aged 18 and over, (2) finished the “Psychiatric Nursing” course at school. The exclusion criteria were as follows: (1) psychiatric nurses and (2) students who had not studied the “Psychiatric Nursing” course. Based on whether participants had experienced psychiatric clinical practice, they were divided into two groups: one group with psychiatric clinical practice and the other group without psychiatric clinical practice.

### 2.2 Study design and data collection

The link of an anonymous online survey, posted on Questionnaire Star Platform (https://www.wjx.cn/), was sent to nurse managers and nursing faculty at the selected institutions from December 2021 to March 2022. They forwarded it to nonpsychiatric nurses and nursing interns who had not been to a psychiatric department or mental health center, respectively. Informed consent, inclusion criteria and exclusion criteria were on the first page of the online survey. When they finished reading this, participants clicked the “Agree” button to indicate their consent to participate in the study, and the questionnaire appeared. In the process of filling in the questionnaire, if there is any missing item, it cannot be submitted, and the system will mark the missing item for participants to fill in. A total of 1355 valid questionnaires were retrieved, excluding 15 questionnaires from nurses working in the mental health center and 16 from nursing interns who had working experience in a psychiatric department or mental health center. Finally, 1324 questionnaires were included in this study. All data can only be obtained online by researchers publishing the questionnaires.

### 2.3 Measurement

#### 2.3.1 General information questionnaire

A Self-administered questionnaire was used to collect participants’ demographic data, personal care experience and psychiatric nursing education. Demographic data included identity (including nurses and nurse interns), age, gender, working department, education level, marital status, only child status and long-term residence. Psychiatric nursing education was identified by the question “What kind of psychiatric nursing education have you received?” Participants should choose “Psychiatric course only” or “Psychiatric course and clinical practice in psychiatric department (at least 4 weeks to 3 months)”. Personal care experience was identified by the answer of “yes” to the question “Have you ever been involved in caring for someone diagnosed with mental disorders (in a nontherapeutic relationship), such as a family member, friend or neighbor”?

#### 2.3.2 Community Attitudes towards the Mentally Ill

The CAMI was used to measure participants’ attitudes towards mental disorders (28). It consists of 40 items, which are rated on a 5-point scale from 1 strongly disagree to 5 strongly agree, depending on how much one agrees or disagrees with the items. It was translated into Chinese in 1998 (29). The CAMI examines four dimensions of attitudes towards mental disorders, namely, authoritarianism, social restrictiveness (SR), benevolence and community mental health ideology (CMHI). Authoritarianism refers to the view that mental disorders need coercive treatment and SR involves the view that patients with mental disorders are a threat to society, which indicates negative attitudes towards mental disorders. The higher the score is, the worse the attitude is. Benevolence reflects sympathy for mental disorders, and the CMHI is about mental health services and the acceptance of mental disorders in the community. Both of them represent positive attitudes towards mental disorders, with higher scores indicating higher acceptance. The CAMI presenting good and acceptable reliability and validity was initially used in community members and then applied to mental health professionals, nurses, students, etc. (30, 31). Previous studies showed that the scale had acceptable reliability (the alpha coefficient for each of the four scales varied from 0.68 to 0.88) and concurrent validity (29, 32).

### 2.4 Data analysis

All analyses were performed using SPSS Statistics 26.0 software. Participants’ general information was analyzed with descriptive statistical methods. Categorical variables were described as frequencies and percentages. Continuous variables were described as the mean and standard deviation (SD). Participants were divided into different age groups on a 10-year line. All variables were subjected to univariable logistic analysis to compare the demographic characteristics, personal care experience and attitudes towards mental disorders between nursing education with and without psychiatric clinical practice. Multivariate linear regression was used to identify characteristics that were associated with the four dimensions of attitudes towards mental disorders. Hierarchical linear regression models were conducted to assess associations between psychiatric nursing clinical practice and attitudes towards mental disorders: authoritarianism, SR, benevolence, CMHI. Model 1 was unadjusted. Model 2 was adjusted for age, education level, marital status and long-term residence. Model 3 was further adjusted for identity and personal care experience. For all analyses, two-sided *p* values less than 0.05 were considered statistically significant.

## 3 Results

### 3.1 Characteristics of participants and their attitudes towards patients with mental disorders

In total, 1324 participants were involved in this study, of whom without psychiatric clinical practice group (those who have had only psychiatric nursing course) mainly consisted of nursing interns whose average age was relatively young. The majority of them were with junior college degrees or below, unmarried and living in rural areas. By contrast, the psychiatric clinical practice group (those who have had both psychiatric nursing course and psychiatric nursing clinical practice) mainly consisted of nurses with older average age. The majority of them had bachelor’s degree and above, married and living in rural areas (presented in **Table 1**). Identity, age, education level, marital status, long-term residence, personal care experience differed significantly between the two groups (*p* < 0.05).

**Table 1:** Comparison of demographic characteristics, personal care experience and attitudes towards mental disorders between nursing education with and without psychiatric clinical practice (N=1324).

Variable	Total N (%)	without PCP N (%)	with PCP N (%)	OR (95%CI)
Identity				
Nurses	603 (45.5%)	39 (6.2%)	564 (81.0%)	ref.
Nursing interns	721 (54.5%)	589 (93.8%)	132 (19.0%)	0.02 (0.01 ~ 0.02)^***^
Age (year)				
16–25	843 (63.7%)	623 (99.2%)	220 (31.6%)	ref.
26–35	346 (26.1%)	4 (0.6%)	342 (49.1%)	242.12 (89.28 ~ 656.58)^***^
36–55	135 (10.2%)	1 (0.2%)	134 (19.3%)	379.46 (52.75 ~ 2729.90)^***^
Gender				
Male	106 (8.0%)	57 (9.1%)	49 (7.0%)	ref.
Female	1218 (92.0%)	571 (90.9%)	647 (93.0%)	1.32 (0.89 ~ 1.96)
Education level				
Junior college and below	739 (55.8%)	549 (86.6%)	190 (26.6%)	ref.
Bachelor’s degree and above	585 (44.2%)	79 (11.9%)	506 (65.2%)	18.51 (13.86 ~ 24.71)^***^
Marital status				
Married	400 (30.2%)	2 (0.3%)	398 (57.2%)	ref.
Unmarried	916 (69.2%)	626 (99.7%)	290 (41.7%)	0.002 (0.001 ~ 0.01)^**^
Divorced or widowed	8 (0.6%)	0 (0%)	8 (1.1%)	NA
Only child				
The only child	519 (39.2%)	230 (36.6%)	289 (41.5%)	ref.
Not the only child	805 (60.8%)	398 (63.4%)	407 (58.5%)	0.81 (0.65 ~ 1.02)
Long-term residence				
Rural area	344 (26.0%)	302 (48.1%)	42 (6.0%)	ref.
Town	255 (19.3%)	178 (28.3%)	77 (11.1%)	3.11 (2.05 ~ 4.73)^***^
Urban area	725 (54.7%)	148 (23.6%)	577 (82.9%)	28.03 (19.37 ~ 40.58)^***^
Personal care experience				
No	1095 (82.7%)	538 (85.7%)	557 (80.0%)	ref.
Yes	229 (17.3%)	90 (14.3%)	139 (20.0%)	1.49 (1.12 ~ 2.00)^**^
**Authoritarianism** (Mean ± SD)		2.78 ± 0.33	2.95 ± 0.38	3.57 (2.60 ~ 4.91)^***^
**Benevolence** (Mean ± SD)		1.95 ± 0.48	1.77 ± 0.43	0.43 (0.34 ~ 0.54)^***^
**SR** (Mean ± SD)		2.76 ± 0.39	2.80 ± 0.41	1.34 (1.02 ~ 1.76)^*^
**CMHI** (Mean ± SD)		2.19 ± 0.41	2.05 ± 0.49	0.51 (0.40 ~ 0.65)^***^

PCP, psychiatric clinical practice; SR, social restrictiveness; CMHI, community mental health ideology; NA, not available. *p<0.05, **p<0.01, ***p<0.001. Reference group is without PCP.

The CAMI subscale scores of nonpsychiatric nurses under two types of psychiatric nursing education showed that the scores of psychiatric clinical practice group were significantly lower than those of without psychiatric clinical practice group in benevolence (OR = 0.43, 95% CI = 0.34 ~ 0.54, *p* < 0.001) and CMHI (OR = 0.51, 95%CI = 0.40 ~ 0.65, *p* < 0.001), and significantly higher in authoritarianism (OR = 3.57, 95%CI = 2.60 ~ 4.91, *p* < 0.001) and SR (OR = 1.34, 95% CI = 1.02 ~ 1.76, *p* = 0.035) (**Table 1**).

### 3.2 Associations between factors and attitudes towards patients with mental disorders among nonpsychiatric nurses

Multivariate linear regression was carried out to test whether the independent variables with the above differences had an impact on the four subscales of CAMI. The results showed that authoritarianism was positively correlated with high education level (B = 0.15, 95%CI = 0.10 ~ 0.21, *p* < 0.001), long residence in urban (B = 0.08, 95%CI = 0.02 ~ 0.14, *p* = 0.009) and personal care experience (B = 0.10, 95%CI = 0.05 ~ 0.15, *p* < 0.001). Benevolence was negatively correlated with psychiatric clinical practice (B = -0.09, 95%CI = -0.17 ~ 0.003, *p* = 0.043), high education level (B = -0.09, 95%CI = -0.16 ~ -0.02, *p* = 0.013), long residence in urban (B = -0.11, 95%CI = -0.19 ~ -0.04, *p* = 0.004) and personal care experience (B = -0.12, 95%CI = -0.19 ~ -0.06, *p* < 0.001). SR was positively correlated with high education level (B = 0.12, 95%CI = 0.06 ~ 0.18, *p* < 0.001), long residence in urban (B = 0.08, 95%CI = 0.01 ~ 0.14, *p* = 0.021) and personal care experience (B = 0.12, 95%CI = 0.06 ~ 0.18, *p* < 0.001). CMHI was negatively correlated with long residence in urban (B = -0.12, 95%CI = -0.19 ~ -0.04, *p* = 0.003), personal care experience (B = -0.12, 95%CI = -0.19 ~ -0.06, *p* < 0.001) and psychiatric clinical practice (B = -0.09, 95%CI = -0.17 ~ -0.01, *p* = 0.027). Results were shown in **Table 2**.

**Table 2:** Associations between factors and attitudes towards mental disorders among nonpsychiatric nurses (N=1324).

Variables	Authoritarianism B (95%CI)	Benevolence B (95%CI)	SR B (95%CI)	CMHI B (95%CI)
Clinical practice				
without PCP	ref.	ref.	ref.	ref.
with PCP	0.04 (-0.03 ~ 0.10)	-0.09 (-0.17 ~ 0.003)^*^	0.01 (-0.06 ~ 0.08)	-0.09 (-0.17 ~ -0.01)^*^
Identity				
Nurses	ref.	ref.	ref.	ref.
Nursing interns	-0.01 (-0.07 ~ 0.06)	-0.01 (-0.09 ~ 0.08)	0.01 (-0.06 ~ 0.08)	0.21 (-0.06 ~ 0.11)
**Age (year)**	0.001 (-0.01 ~ 0.01)	-0.002 (-0.008 ~ 0.005)	-0.003 (-0.01 ~ 0.003)	0.001 (-0.01 ~ 0.01)
Education level				
Junior college and below	ref.	ref.	ref.	ref.
Bachelor’s degree and above	0.15 (0.10 ~ 0.21)^***^	-0.09 (-0.16 ~ -0.02)^*^	0.12 (0.06 ~ 0.18)^***^	-0.05 (-0.12 ~ 0.02)
Marital status				
Married	ref.	ref.	ref.	ref.
Unmarried	0.03 (-0.05 ~ 0.10)	-0.04 (-0.13 ~ 0.05)	0.07 (-0.01 ~ 0.16)	-0.09 (-0.18 ~ 0.003)
Divorced or widowed	-0.21 (-0.46 ~ 0.03)	0.04 (-0.28 ~ 0.35)	-0.30 (-0.57 ~ -0.02)^*^	0.39 (0.08 ~ 0.71)^*^
Long-term residence				
Rural area	ref.	ref.	ref.	ref.
Town	0.07 (0.01 ~ 0.13)^*^	-0.13 (-0.21 ~ -0.06)^**^	0.04 (-0.02 ~ -0.11)	-0.10 (-0.17 ~ -0.03)^**^
Urban area	0.08 (0.02 ~ 0.14)^**^	-0.11 (-0.19 ~ -0.04)^**^	0.08 (0.01 ~ 0.14)^*^	-0.12 (-0.19 ~ -0.04)^**^
Personal care experience				
No	ref.	ref.	ref.	ref.
Yes	0.10 (0.05 ~ 0.15)^***^	-0.12 (-0.19 ~ -0.06)^***^	0.12 (0.06 ~ 0.18)^***^	-0.12 (-0.19 ~ -0.06)^***^

PCP, psychiatric clinical practice; SR, social restrictiveness; CMHI, community mental health ideology; *p<0.05, **p<0.01, ***p<0.001.

### 3.3 Relationship between CAMI subscale scores and two types of psychiatric nursing education among nonpsychiatric nurses

**Table 3** showed the results from the hierarchical linear regression models for the relationship between CAMI subscale scores and two types of psychiatric nursing education in nonpsychiatric nurse burden while controlling for contextual variables. Psychiatric clinical practice was initially associated with higher score of authoritarianism (B = 0.16, 95%CI = 0.13 ~ 0.20, *p* < 0.001) and SR (B = 0.05, 95%CI = 0.003 ~ 0.09, *p* = 0.035), lower score of benevolence (B = -0.18, 95%CI = -0.23 ~ -0.13, *p* < 0.001) and CMHI (B = -0.14, 95%CI = -0.19 ~ -0.09, *p* < 0.001) (Model 1). As for the initial associations between psychiatric clinical practice status and authoritarianism, SR disappeared after adjusting for age, education level, marital status and long-term residence (Model 2), as well as further adjusted for identity and personal care experience (Model 3).

**Table 3:** Relationship between CAMI subscale scores and two types of psychiatric nursing education among nonpsychiatric nurses (N=1324).

		Authoritarianism B (95%CI)	Benevolence B (95%CI)	SR B (95%CI)	CMHI B (95%CI)
**Model 1**	without PCP	ref.	ref.	ref.	ref.
	with PCP	0.16 (0.13 ~ 0.20)^***^	-0.18 (-0.23 ~ -0.13)^***^	0.05 (0.003 ~ 0.09)^*^	-0.14 (-0.19 ~ -0.09)^***^
**Model 2**	without PCP	ref.	ref.	ref.	ref.
	with PCP	0.04 (-0.02 ~ 0.10)	-0.08 (-0.16 ~ -0.01)^*^	0.01 (-0.06 ~ 0.07)	-0.10 (-0.18 ~ -0.03)^**^
**Model 3**	without PCP	ref.	ref.	ref.	ref.
	with PCP	0.04 (-0.03 ~ 0.10)	-0.09 (-0.17 ~ -0.003)^*^	0.01 (-0.06 ~ 0.08)	-0.09 (-0.17 ~ -0.01)^*^

PCP, psychiatric clinical practice. SR, social restrictiveness; CMHI, community mental health ideology; Model 1, unadjusted linear regression model. Model 2, adjusts for age, education level, marital status and long-term residence. Model 3, model 2 with additional adjustment for identity and personal care experience. *p<0.05, **p<0.01, ***p<0.001.

## 4 Discussion

To our knowledge, this study is one of the few to investigate the attitudes of nonpsychiatric nurses (nonpsychiatric nurses and nursing interns) towards mental disorders and factors associated with their attitudes in China. Although we originally found that those taking psychiatric clinical practice were more likely to exhibit authoritarianism and SR, and less likely to exhibit benevolence and CMHI towards mental disorders than those without psychiatric clinical practice. Only benevolence and CMHI were still correlated with psychiatric clinical practice after adjusting confounding factors. In addition, education level, long-term residence, personal care experience and psychiatric nursing education were associated with different dimensions of attitudes to mental disorders.

This study showed that the nonpsychiatric nurses who received psychiatric nursing clinical practice in the psychiatric department had less empathy and community acceptance towards mental disorders than those who did not. This was inconsistent with previous findings. Some previous studies found that psychiatric nursing clinical practice could positively affect the attitudes of nursing students towards mental disorders (33–36). However, others reported an negative effect, which suggested that the effect of clinical practice still needed to be explored (9, 37). Such inconsistency might be caused by different states of the contacted patients (38, 39). The systematic review emphasized that interacting with stable patients (such as in a state of recovery) in a relatively safe clinical environment helped to challenge the stereotypes of mental disorders (40). However, the states of contacted patients of this study were not restricted. Therefore, future studies should investigate whether the state of patients will impact the effects of psychiatric nursing clinical practice on the attitudes of nursing students to give insight into the reform of psychiatric nursing education.

In addition, the timing of assessment of attitudes towards mental disorders might also contribute to the inconsistency (41). The systematic review found that the effectiveness of clinical practice in promoting health care students’ attitudes towards mental disorders was short-term, disappearing in one-year follow-up (40). The assessment of attitudes towards mental disorders were conducted years after the completion of psychiatric nursing clinical practice for nonpsychiatric nurses. Therefore, future studies should assess the short-term and long-term effects of psychiatric nursing clinical practice. Moreover, to sustainably improve nonpsychiatric nurses’ attitudes towards mental disorders, psychiatric nursing education should be added to their professional education.

Our study found that nonpsychiatric nurses with higher education levels were more likely to agree that patients with mental disorders were inferior, should be coerced into treatment or were a threat to society and were less likely to empathize with such groups. This was in contrast to most previous studies: with the improvement in nursing education level, mental health literacy also gradually improved (42, 43). However, a recent study on the care behavior of emergency department nurses towards mental disorders found a significant inverse correlation between level of education and care behavior, which is similar to our results (44). This result may be due to the fact that nurses with higher levels of education, who play more administrative roles, have less contact time with mental disorders (45).

This study also showed that individuals who lived for a long time in rural areas had significantly better attitudes towards mental disorders than people who lived in urban areas. This was supported by the results of previous studies showing that medical students from rural areas had a better attitude to patients with mental disorders than those from urban areas (46, 47). Mental health services and facilities in urban communities are more mature and comprehensive, and there is greater dissemination of knowledge about mental disorders (48). However, the media in China are in general filled with negative images of psychiatric patients, which can powerfully influence the urban residents’ attitudes towards mental disorders (49). In addition, there is a deep-rooted family collectivism in China’s rural areas, where families are likely to come together to take care of the sick (50), which may be related to a reduction in stigma.

Furthermore, we found that nonpsychiatric nurses who had been involved in the care for mental disorders on a personal level (as family members, friends or neighbors) had more negative attitudes towards these people; that is, they considered people diagnosed with mental disorders to be treated compulsively, a threat to society, and had less empathy and acceptance towards them. The reason for this phenomenon may be related to the personal care situation in which the nonpsychiatric nursing groups were involved. Nonpsychiatric nursing groups do not care for mental disorders on a full-time basis but as support to control their abnormal behaviors or seek medical assistance when their mental condition deteriorates, which might provide an inadequate and incorrect view of mental disorders and reinforce stereotypes. This suggests that the quality of contact with people diagnosed with mental disorders greatly affects people’s attitudes towards mental disorders (51, 52). Not having extensive professional practice in dealing with the symptoms of mental disorders can be frustrating and resistant to these people, which may lead to the belief that mental disorders are unpredictable and untreatable (53). Therefore, clinical practice lecturers should be aware of students’ relevant nursing experiences at the beginning of clinical practice, provide them with counseling and support, and help them overcome negative thoughts through positive practice experiences.

## 5 Strengths and limitations of the study

The study is the first in China to focus on the influence of psychiatric clinical practice on the attitudes of nonpsychiatric nurses towards mental disorders. The results revealed that the psychiatric nursing clinical practice of nonpsychiatric nurses did not improve the discrimination of nonpsychiatric nurses towards mental disorders, suggesting that psychiatric nursing clinical practice should be further strengthened. Meanwhile, this study is one of the few studies researching the factors that influence the stigma of mental disorders in nonpsychiatric nurses, providing new insights into further studies in the field. However, there were also some limitations to this study. First, although this was a cross-sectional study and couldn’t explain the causal relationship between variables, we collected questionnaires from different centers and used reliable evaluation tools to minimize the bias in horizontal surveys. Second, the samples of this study were all from cities in southwest China, so the generalization of the conclusion needs to be done with caution and may not represent the whole country. Third, the total number and proportion of eligible nurses and nursing interns within selected institutions could not be determined because of the convenience sampling method in each institution, which might lead to bias in some degree.

## 6 Conclusion

This study showed that nonpsychiatric nurses who had taken both psychiatric nursing courses and psychiatric nursing clinical practice had a more negative attitude towards mental disorders than those who had taken only psychiatric nursing courses. Besides, education level, personal care experience and long-term residence were associated with attitudes towards mental disorders among nonpsychiatric nurses in China. These suggested that psychiatric nursing clinical practice education should be improved, such as offering them a contact with patients during the remission period of mental disorders, and adding psychiatric nursing to the vocational education for nonpsychiatric nurses. The quality and the duration of nursing clinical practice might affect the level of stigma, so we should actively explore better psychiatric nursing clinical practice models. During the clinical practice process, emphasis should be placed on lessening the stigma of mental disorders among nursing interns, and active guidance should be given to them on correctly understanding the negative effects of mental disorders, aiming to reduce the discrimination towards mental disorders among nonpsychiatric nurses. Further research could take longitudinal studies to explore the association between psychiatric nursing clinical practice and the attitudes of nonpsychiatric nurses towards mental disorders. And it is necessary to explore practical strategies to optimize the psychiatric nursing clinical practice experience of nonpsychiatric nurses, thus to improve their attitudes towards mental disorders.

## Data availability statement

The raw data supporting the conclusions of this article will be made available by the authors, without undue reservation.

## Ethics statement

The studies involving humans were approved by the Biomedical Ethics Committee of West China Hospital, Sichuan University (ID: 2019–686). Informed consent was obtained from all participants before data collection. The studies were conducted in accordance with the local legislation and institutional requirements. The participants provided their written informed consent to participate in this study.

## Author contributions

Q-KW: Data curation, Formal analysis, Writing – original draft. XW: Investigation, Methodology, Project administration, Writing – original draft, Resources, Validation. Y-JQ: Supervision, Validation, Writing – review & editing, Formal analysis. W-XB: Validation, Writing – review & editing, Investigation. X-CC: Data curation, Formal analysis, Project administration, Writing – original draft, Conceptualization, Supervision, Validation. J-JX: Conceptualization, Data curation, Investigation, Project administration, Supervision, Writing – review & editing, Validation.
